# Supplementary material for: Analyzing the impact of socioeconomic indicators on gender inequality in Sri Lanka: A machine learning-based approach
Source: PLoS One. 2024 Dec 26;19(12):e0312395. doi: 10.1371/journal.pone.0312395 (PMC11671024; doi:10.1371/journal.pone.0312395)
Supplement: S1 Table — (PDF) [file pone.0312395.s001.pdf]

S1 Table: Description of variables in the model

Code	Variable Name and Description
GDS	GDP based on purchasing power parity, the share of the world.<br>Purchasing Power Parity (PPP) weights are individual countries' share of total World gross domestic product at purchasing power parities.
GDC	GDP per capita at current prices; Total value at current prices of final goods and services produced within a country during a specified time period divided by the average population for the same one year.
GDP	GDP, current prices; Total value at current prices of final goods and services produced within a country during a specified time period; one year.
GEX	Government expenditure as a percent of GDP
GPB	Government primary balance as a percent of GDP
GPE	Government primary expenditure as a percent of GDP
GRV	Government revenue as a percent of GDP
UER	The number of unemployed persons as a percentage of the total labor force.
