# Supplementary material for: Analyzing the impact of socioeconomic indicators on gender inequality in Sri Lanka: A machine learning-based approach
Source: PLoS One. 2024 Dec 26;19(12):e0312395. doi: 10.1371/journal.pone.0312395 (PMC11671024; doi:10.1371/journal.pone.0312395)
Supplement: Attachment — Submitted filename: Response_to_Reviewer_2_Comments.pdf [file pone.0312395.s002.pdf]

Response letter for manuscript PONE-D-24-24357

August 21, 2024

Reviwer 2 Comments

The paper explores the relationship between different socioeconomic factors and the gender inequality index (GII) in Sri Lanka. It uses different methodologies and compares them with each other to determine which one is the most appropriate, highlighting the use of machine learning (ML) techniques. The topic of the study is of great interest, is very topical and adds value to the existing literature. However, I believe that the paper needs to be restructured and needs to answer the research question more precisely. In the following paragraphs I detail my suggestions. I hope that the authors will find my comments useful in improving the paper.

1 **Contribution: I consider the contribution of the paper to be adequate. The study addresses an interesting topic from different approaches. However, I think it lacks further theoretical development to support the research question. The content of the introduction is adequate, but the main hypothesis of the study should not be stated in the introduction and the variables should not be explained.**

Authors' responses:

Thank you for your suggestion. We have restricted and rewrote the Introduction by removing the wording of the main hypothesis and the variable introduction. The updated content is highlighted in the updated manuscript for your reference.

2 **Literature review: despite being a fundamentally practical paper, the literature review section is scarce. I consider this to be the paper's biggest problem. There is a lack of a definition of the objective, a lack of support for the research with previous literature and a lack of specification of what is to be analysed in a concrete way. There is no theoretical underpinning throughout the paper to understand the relationships that are subsequently explained in the results. Hypotheses should be stated here. Moreover, there should be a separate hypothesis for each socioeconomic factor as the effect is different for each of**

them. On the other hand, the gender inequality index, which is the important variable in the study, is not justified, nor is it explained how it is calculated or its components.

Authors' responses:
Thank you for the suggestion. We rewrote the literature review to address your comments and enriched the section by including pass studies that are more relevant to our work.

Moreover, we addressed the main hypothesis here and added separate explanations for each variable used in the study to give a clear understanding of the work to the reader.

The updated content is highlighted in the manuscript for your reference.

3 **Methodology and results: The methodology section is good. The procedure is clearly detailed, making it easy for the reader to understand the paper. As for the data and variables, the source of the data is explained in detail, but the detailed explanation of the variables is not as it was done in the introduction.**

Authors' responses:
Thank you for the comment. We have updated the methodology section of the manuscript by adding the following details about the variables used in this work.

The key variables used in this analysis are detailed below, including their definitions, measurement methods, and sources.

a) *Gender Inequality Index*

The GII is the dependent variable in this study. It represents gender-based disparities in three critical dimensions: reproductive health, empowerment, and labor market participation. The GII ranges from 0, indicating perfect gender equality, to 1, reflecting complete gender inequality. This index is a composite measure that captures the extent of inequality between men and women in these areas and is sourced from the United Nations Development Programme (UNDP).

b) *Gross Domestic Product*

GDP is a key economic indicator used in the study, representing the total value of goods and services produced within a country in a given period, adjusted for inflation (real GDP). It is a measure of overall economic performance and is expressed in current prices. Higher GDP levels are generally associated with improved economic conditions and potentially lower gender inequality. The GDP data used in this study is obtained from the World Bank.

c) *GDP Per Capita*

GDC is another crucial economic indicator, calculated by dividing the GDP by the total population. This variable provides insight into the average economic output per person, reflecting the standard of living in the country. Higher GDP per capita often correlates with better access to resources and opportunities, which can influence gender inequality. The data for GDP per capita is sourced from IMF.

d) *Government Expenditure*

GEX refers to the total government spending as a percentage of GDP, including consumption, investment, and transfer payments. This variable is critical for understanding the role of fiscal policy in shaping gender inequality. Higher government expenditure might lead to improved public services, which can reduce gender disparities, but it could also exacerbate inequality if not properly targeted. The GEX data is sourced from the IMF.

e) *Government Revenue*

GRV is the income generated by the government from taxes and other sources, expressed as a percentage of GDP. It is a key indicator of the government's capacity to finance public services, which can impact gender equality by providing resources for education, healthcare, and social programs. The GRV data used in this study is sourced from the IMF.

f) *Government Primary Balance*

GPB is the government's fiscal balance, excluding interest payments on outstanding debt, expressed as a percentage of GDP. A positive primary balance indicates that the government is generating more revenue than spending (excluding debt interest), which may provide more room for gender-focused investments. The data for GPB is sourced from the IMF.

g) *Government Primary Expenditure*

GPE measures the government's spending on goods, services, and transfer payments, excluding interest payments on debt, as a percentage of GDP. This variable helps in understanding the government's spending priorities and its potential impact on gender inequality. The data for GPE is obtained from the IMF.

h) *Unemployment Rate*

UER represents the percentage of the labor force that is unemployed and actively seeking employment. It is a key indicator of labor market health and can have significant implications for gender inequality, particularly in terms of access to economic opportunities. Higher unemployment rates may exacerbate gender disparities, especially if women are disproportionately affected. The UER data used in this study is sourced from the World Bank.

The updated content is highlighted in the manuscript.

4 In the results section, the empirical analysis is very interesting and detailed. The graphs and tables facilitate the understanding of the results. The different results are much more detailed taking into account the different methodologies used than the effect of the different socioeconomic factors. At no point is a test of the previously stated hypothesis carried out.

Authors' responses:
Thank you for the comment. We have modified the results and discussion section by adding the following section to validate our results by Hypothesis testing.

Hypothesis Testing and Empirical Analysis

In this study, the primary objective was to investigate the relationship between key economic indicators and GII in Sri Lanka. To achie ve this, we formulated hypotheses that certain economic factors—such as GDP, government expenditure, and unemployment rate—would have significant predictive power in explaining variations in GII.

Testing of Hypotheses Using ML Models

The hypotheses were tested using multiple ML models, including Decision Trees, Ensemble Methods, linear regression, SVM, and Gaussian Process Regression. These models were chosen for their ability to capture complex relationships between the independent variables (economic indicators) and the dependent variable (GII).

The results of the ML models provided strong evidence supporting our hypotheses. For instance, the Fine Tree regression model, with an R^2 value of 0.975, indicated that the selected economic indicators collectively explain 97.5% of the variance in GII. This high level of explained variance suggests that the economic indicators we hypothesized as significant predictors indeed play a crucial role in determining gender inequality in Sri Lanka.

Validation of Hypotheses with SHAP Analysis

We utilized SHAP values to validate the results further and understand the contribution of each economic indicator to the GII.

The SHAP analysis revealed that GDP and GDC were among the most significant predictors of GII, with SHAP values of +2.57 and +1.01, respectively. These findings are consistent with our hypothesis that economic growth and development are inversely related to gender inequality. The positive SHAP values indicate that increases in GDP and GDC contribute significantly to reducing GII, thus supporting the idea that economic prosperity is linked to greater gender equality.

On the other hand, GEX and UER also showed noticeable impacts on GII, albeit with different directions of influence. GEX had a positive SHAP value, suggesting that higher government spending might exacerbate gender inequality if not properly targeted, while UER's positive SHAP value indicates that higher unemployment rates are associated with higher GII, aligning with our hypothesis.

Hypothesis Testing

The combination of ML model results and SHAP analysis provides robust support for our initial hypotheses. The empirical analysis demonstrates that the economic indicators selected are not only correlated with gender inequality but also play significant roles in shaping it. By thoroughly testing these hypotheses through advanced statistical and machine learning techniques, our study contributes valuable insights into the socioeconomic factors that influence gender inequality in Sri Lanka.

The updated content is highlighted in the manuscript for your reference.

5 Conclusions: I consider that the conclusions are correct. The results are correctly explained and the role of gender in this study, the causes and effects are argued for the first time in the document. The implications of the study in a field beyond the academic one are explained.

Authors' responses:
Thank you for your kind comment on the conclusion.

•

Note: The updated content is highlighted in the manuscript for your reference.
The authors appreciate all of the insightful comments from the reviewer. We worked hard to be responsive to them. Thank you for taking the time and energy to help us improve the paper.

Thank you.
