# Supplementary material for: Analyzing the impact of socioeconomic indicators on gender inequality in Sri Lanka: A machine learning-based approach
Source: PLoS One. 2024 Dec 26;19(12):e0312395. doi: 10.1371/journal.pone.0312395 (PMC11671024; doi:10.1371/journal.pone.0312395)
Supplement: Attachment — Submitted filename: GII___Response_to_Reviewer_2_r2_Comments.pdf [file pone.0312395.s003.pdf]

Response letter for manuscript PONE-D-24-24357R1]

October 3, 2024

Reviwer 2 Comments
Thank you for reviewing the manuscript and addressing my comments in the first report. I believe that the document is now more complete, but I feel that there are still areas for improvement. I detail my suggestions in the following paragraphs. I hope that the authors will find my comments useful in improving the paper. I still find the literature review lacking. The objective is now much clearer and the hypotheses are more specific, but theoretical support is still lacking. There is no mention of any theory being followed to formulate either the objective or the hypotheses. I reiterate the need to support the research with previous literature to substantiate the paper. The changes made in the introduction do not seem to me to satisfy all the comments made in the first report, so I consider that it is necessary to restructure this section again (see the comments of the literature review of the first report).

Authors' responses:

Thank you for your constructive feedback. In response to your suggestions, we have significantly enhanced the literature review by incorporating relevant theoretical frameworks to better support the research objective and hypotheses. We have also restructured the introduction, aligning it more closely with the comments from the first report, ensuring a clearer and more comprehensive presentation of the research background. We trust these revisions address your concerns and contribute to the overall improvement of the paper.
The updated content is highlighted in yellow for your reference.

-

The authors appreciate all of the insightful comments from the reviewer. We worked hard to be responsive to them. Thank you for taking the time and energy to help us improve the

paper.

Thank you.
